# How to treat orthostatic tremor – Cohort study and systematic review

**DOI:** 10.1016/j.prdoa.2025.100318

**Published:** 2025-04-01

**Authors:** W.A. Babeliowsky, M.A. Meulepas, A.W.G. Buijink, R.M.A. de Bie, A.F. van Rootselaar

**Affiliations:** a Department of Neurology and Clinical Neurophysiology, Amsterdam University Medical Center, Amsterdam Neuroscience, University of Amsterdam, Amsterdam, the Netherlands; b Amsterdam Neuroscience, Neurodegeneration, Meibergdreef 9, 1105 AZ Amsterdam, the Netherlands

**Keywords:** Orthostatic Tremor, Treatment, Pharmacological, Surgical, Systematic Review, Cohort Study

## Abstract

**Introduction:**

Orthostatic tremor (OT) is a highly disabling condition. Effectiveness of treatments is largely unverified and no treatment guideline exists. The objective of this study is to evaluate the efficacy and adverse effects of treatments used for OT.

**Methods:**

Patients from our online OT-registry were compared to findings from a systematic search in PUBMED. The search included original articles describing clinical features in human primary OT (electromyography peak frequency ≥ 13 Hz), in English or Dutch, and published after 1985. Outcome measures were prescribed treatments and their efficacy (percentage of patients per treatment with a reduction of tremor symptoms) and adverse effects.

**Results:**

A total of 78 primary OT patients in the Netherlands and 613 OT patients from 74 articles, mostly case reports and cohort studies, were included. In the cohort from the Netherlands, perampanel showed both the highest efficacy and adverse effects. In the literature, clonazepam showed the highest efficacy, while data on adverse effects was lacking. Overall, pharmacological treatments were reported often to be insufficient. Bilateral deep brain stimulation (DBS) was reported to be effective in the majority of patients, without a higher prevalence of adverse effects.

**Conclusion:**

Pharmacological treatment is sufficient in some patients, although overall efficacy is limited and often accompanied by adverse effects. Perampanel and clonazepam are first choice treatments. Surgical interventions seem suitable alternatives for medication resistant patients, although this is based on small studies. Future studies should use diagnostic criteria, standardized outcome scales and good study designs, to increase reliability of findings and comparability.

## Highlights

• Perampanel seems the most effective drug for OT but has a high side-effect rate.
• Perampanel and clonazepam are first choice treatment in OT.
• Surgical interventions provide consistent positive outcomes.
• Surgical treatments are suitable alternatives for medication-resistant OT patients.

Orthostatic tremor (OT) is an orphan disease first described in 1970, and officially named in 1984 [1], [2]. OT manifest as a distinctive tremor of ≥ 13 Hz, primarily affecting the lower extremities during stance, provoking sensations of instability and apprehension of falling. Consequently, patients often seek support, walk, or sit down, which limits their daily activities [2], [3]. Diagnosis relies on surface electromyography (EMG) to the lower limb muscles, revealing the characteristic rhythmic bursts during stance and absent in rest [4], [5], [6], [7], [8]. OT predominantly affects middle-aged to elderly individuals, exhibits a female predilection, and often progresses to a state of (almost) complete inability to stand still [9], [10].

Despite its profound impact on quality of life, OT lacks targeted treatment options let alone a definitive cure. Current treatment options are limited, predominantly relying on medications repurposed from other conditions and administered off-label. Prescribed pharmacological agents include benzodiazepines, anticonvulsants, β-blockers, and antiparkinsonian drugs [11], [12], [13], [14]. In addition, deep brain stimulation (DBS) and spinal cord stimulation (SCS) have been reported in the literature as treatment options for OT, with the primary DBS target for OT being the ventral intermediate nucleus of the thalamus (Vim) followed by the caudal zona incerta (cZI) [15], [16], [17]. More recently transcutaneous electrical stimulation have been tried for OT with promising results [18]. However, a treatment guideline does not exist.

It is known that the clinical effect of these medical and surgical treatments can vary substantially between patients, while their true efficacy remains largely unexplored, with current knowledge based on case reports and case-series [11], [13], [16], [19], [20]. One cohort study has investigated the efficacy of OT treatment, but recent developments, such as the off-label use of perampanel, were not included [11]. Furthermore, the occurrence of adverse effects has not been thoroughly reported on, and novel and experimental treatment options have not been reviewed. A systematic review, evaluating all treatment modalities and their (potential) effect on tremor and adverse effects, is lacking the literature.

This study aims to evaluate the efficacy of treatments for OT based on symptom reduction and their associated adverse effects, for pharmacological, surgical, and experimental treatment options. We report novel data from a large OT-cohort and discuss results of a systematic literature search. Results may help to refine treatment strategies, thereby reducing the impact of this debilitating disorder and may help to initiate new research by defining new hypothesis for research.

## 1 Methods

### 1.1 Primary orthostatic tremor inclusion criteria and case selection

Participants from the cohort and cases from the literature had to fulfill a diagnosis of primary OT following the Movement Disorder Consensus Criteria to be included [8]. OT-plus and secondary cases were excluded, as well as double cases (cases reported more than once).

### 1.2 Participants cohort

We included all patients from our online registry (Dutch OT Patient Support Group and Amsterdam University Medical Centers (Amsterdam UMC)), who had: 1) a EMG confirmed diagnosis of primary OT [8], 2) completed the Dutch OT-questionnaire between May 2014 and July 2023, and 3) gave informed consent. All patients provided written consent for their data to be used for this study, although the Institutional Review Board (IRB) of Amsterdam UMC waived the need for specific consent for scientific use of this data.

### 1.3 Data collection and extraction cohort from the Netherlands

Data was extracted from the completed Dutch OT-questionnaires (Supplementary File 1). This questionnaire, developed (2014) and distributed in collaboration with the Dutch OT Patient Support Group, includes questions regarding current and previous treatments, the efficacy, and the adverse effects [21]. Patients had easy and independent access through the website of the Dutch OT Patient Support Group, either via internet searches or via their healthcare providers. Additionally, it was distributed annually during the OT patient day. Patients under the care of Amsterdam UMC, received it annually since 2018 as part of clinical protocols, and this data was supplemented by clinical data extracted from electronic patient files.

### 1.4 Literature search

A systematic literature search was performed in PUBMED on March 31st 2023, using the following search term: “Primary orthostatic tremor”[Supplementary Concept] OR “orthostatic tremor”[tiab] OR “shaky leg syndrome”. Retrieved articles were supplemented by articles from their reference lists. Criteria for exclusion of articles were: study not about the clinical spectrum, review, not written in English or Dutch, studies with patients from the current cohort from the Netherlands, written before 1985 (OT was introduced in 1984), and not concerning humans. Quality of included studies was assessed using the JBI’s critical appraisal tools [22].

### 1.5 Baseline characteristics, outcome measures and analysis

The following baseline characteristics was collected: sex, age at onset, diagnostic delay, and tremor frequency.

All previous and current treatments (i.e., pharmacological, surgical, and other) were documented. Pharmacological treatments were subclassified in the different subclasses of drugs, and based on the latest available questionnaires it was noted whether patients were on monotherapy or combination therapy. Per treatment, efficacy (i.e., reduction of tremor symptoms; categorized as sufficient benefit, no effect, or effect unknown) was assessed, and the adverse effects (yes, no, or unknown). Expressed as percentage of patients per treatment.

Descriptive analyses were used to describe the result obtained in this study. Results from the cohort from the Netherlands and OT patients from the literature are presented separately and compared. Missing values are not replaced. Number of patients with known entries are reported (n = xx).

## 2 Results

### 2.1 Study population

#### 2.1.1 Cohort from the Netherlands

**Fig. 1:**
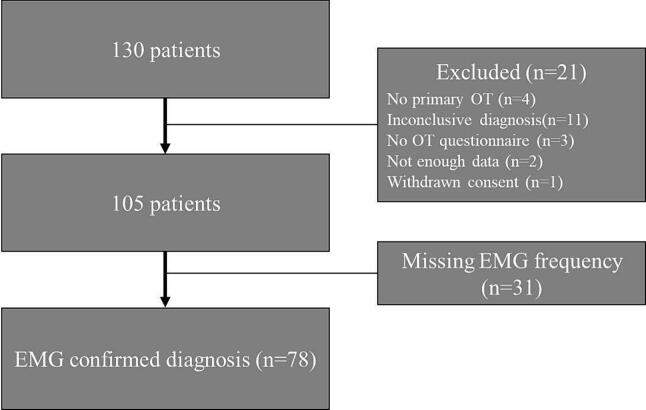
Flow chart of the selection of patients from the cohort from the Netherlands.

Of 130 patients registered in the online database 52 patients were excluded (Fig. 1). Of the remaining 78 patients, 53 (67.9 %) were female, see baseline characteristics (Table 1).

**Table 1:** Demographic data.

**Outcome measure**	**Dutch cohort**				**Literature search**							
	**EMG confirmed OT**	**n**	**EMG reported OT**	**n**	**Case reports**	**n**	**Case series**	**n**	**Case-control**	**n**	**Trials**	**n**
**Age at diagnosis**	62.3 ± 11.1	77	63.0 ± 8.5	30	−	−	−	−	−	−	−	−
**Age at onset**	54.6 ± 12.8	76	57.0 ± 10.1	30	55.4	65	64.1	369	51.9	93	−	−
**Diagnostic delay**	8.5 ± 7.7	77	7.2 ± 11.2	30	6.0	21	7.6	237	9.0	45	−	−

Values are given in mean ± SD for the Dutch cohort and in mean for the literature search.

Abbreviations: n = number of subjects, - = No data available.

Mean follow-up cohort was 31.9 months (range 0–106.5) with a mean of 3.4 (range 1–10) completed questionnaires per patient. Three patients died during follow-up.

#### 2.1.2 Literature

**Fig. 2:**
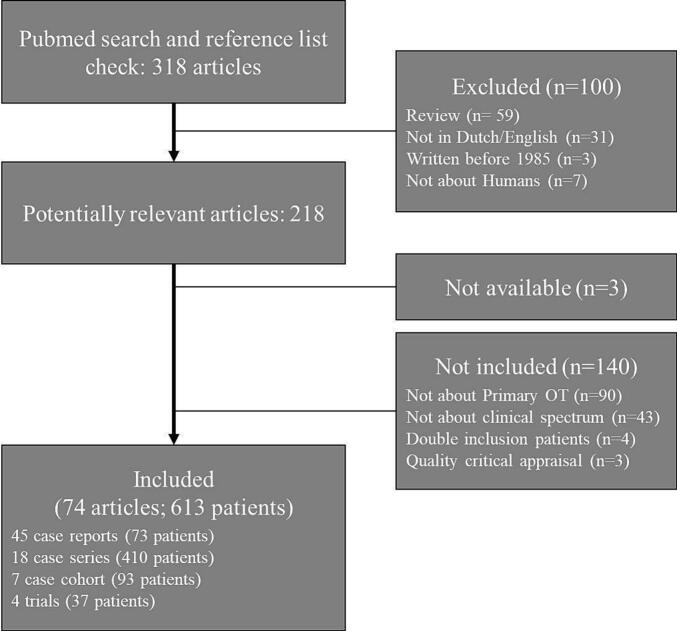
Flow chart of the selection of reviewed articles.

The literature search revealed 77 relevant articles. Overall, the level of evidence and scientific quality of the included articles was rated low to medium. Three articles were excluded based on the critical appraisal, resulting in 74 articles (entailing 613 OT patients; 417 (68.0 %) females) including 45 case reports (73 patients; 37 (50.7 %) female), 18 case-series (410 patients; 281 (68.5 %) female), seven cohort studies (93 patients; 72 (77.4 %) female), and four therapeutic trials (37 individual patients; 27 (73 %) female) (Fig. 2). See Supplementary Data for an overview of the included articles and Table 1 for baseline characteristics.

Because of variations in study designs, methodologies, and reported outcomes, a narrative synthesis was conducted to summarize the findings of the included studies.

### 2.2 Pharmacological treatment

#### 2.2.1 Cohort from the Netherlands

In the cohort from the Netherlands 63 out of 75 patients (three missing) reported (previously) using one or more pharmacological treatments, with twelve patients not taking any pharmacological treatment at any point. In total, thirteen different drugs have been reported, divided over six pharmacological subclasses (Table 2). Drug dosages were mostly missing. Most reported drugs were clonazepam (25.8 %; n = 57), perampanel (19.9 %; n = 44), gabapentin (19.0 %; n = 42), and propranolol (14.0 %; n = 31). The anticonvulsants as a group were most often prescribed, and were reported most efficacious. The most efficacious was perampanel (68.2 %; n = 30 out of 44), followed by clonazepam (47.4 %; n = 27 out of 57), propranolol (35.5 %; n = 11 out of 31), and gabapentin (31.0 %; n = 13 out of 42). Out of 221 entries in total, patients reported a sufficient benefit in 41.2 % (91 entries). In 51.6 % (114 entries), patients did not experience any effect, and in 7.2 % (16 entries), effect was unknown. A total of 57 patients were on monotherapy, fourteen were on combination therapy, 48 had discontinued all medication, and the treatment status of one patient was unclear.

**Table 2:** Treatment effect cohort from the Netherlandss.

**Medication**	**No. of cases**	**Treatment effect**			**Side-effects**		
		**None**	**Sufficient benefit**	**Unknown**	**Yes**	**No**	**Unknown**
***Benzodiazepines***							
**Clonazepam**	57	28	27	2	37	15	5
**Diazepam**	4	3	1	−	3	−	1
**Oxazepam**	9	2	2	5	2	1	6
**All benzodiazepines**	70	33	30	7	42	16	12
***Anticonvulsants***							
**Gabapentin**	42	27	13	2	26	10	6
**Primidone**	9	6	3	−	4	1	4
**Topiramate**	4	2	2	−	2	1	1
**Pregabalin**	3	1	−	2	2	−	1
**Carbamazepine**	1	1	−	−	1	−	−
**Perampanel**	44	13	30	1	33	9	2
**All anticonvulsants**	103	50	48	5	68	21	14
***β-Blockers***							
**Propranolol**	31	18	11	2	14	6	11
***Antiparkinsonian***							
**Levodopa/carbidopa**	15	12	1	2	9	1	5
***Antidepressants/*** ***anxiolytics***							
**Mirtazapine**	1	−	1	−	−	1	−
**Sertraline**	1	1	−	−	−	−	1
**All antidepressants/** **anxiolytics**	2	1	1	−	−	1	1

Abbreviations: No. = number of, − = No data available.

Data on adverse effects was available for twelve of the thirteen prescribed drugs (Table 2). Drugs with the most reported adverse effects were perampanel (75.0 %; n = 33 out of 44), clonazepam (64.9 %; n = 37 out of 57), gabapentin (61.9 %; n = 26 out of 42), and propranolol (45.2 %; n = 14 out of 31). Among the 221 entries in total, patients reported adverse effects in 60.2 % (133 entries). In 20.4 % (45 entries), patients did not experience adverse effects, and in 19.5 % (43 entries), it was unknown whether adverse effects occurred. This included the four drugs with missing data on adverse effects.

#### 2.2.2 Literature

In the literature, pharmacological treatment was reported in 471 patients: 63 patients from case reports, (six missing), 304 patients from case-series (97 missing), 78 patient from case-control studies, and 26 patients from trials. Among these, 39 (four from case reports, nine from case-series, 15 from case-control studies, and 11 from trials) patients did not receive any pharmacological treatment. A total of 57 different drugs categorized into eight pharmacological subclasses (Supplementary Table 1), have been reported. Drug dosages were mostly missing. Clonazepam was effective in 40.3 % of cases (n = 125 out of 310 patients), gabapentin in 26.9 % (n = 36 out of 134 patients), propranolol in 23.8 % (n = 20 out of 84 patients), and primidone in 20.8 % (n = 20 out of 96 patients). Out of the 948 total entries, patients reported sufficient benefit in 27.3 % (259 instances), while 52.6 % (499 instances) reported no effect, and in 20.0 % (190 instances) the effect was unknown. Benzodiazepines were the most frequently prescribed drug class and were also reported to be most effective.

In the majority of studies reported in the literature, adverse effects were not mentioned; it was unclear whether adverse effects occurred in 851 out of 954 entries (89.2 %).

### 2.3 Surgical treatment

#### 2.3.1 Cohort from the Netherlands

Of the nine patients who underwent bilateral Vim-DBS surgery in the Amsterdam UMC, seven experienced sufficient benefit, demonstrated by increased standing time, sustained during long-term follow-up. Assessments for these patients were not blinded, and outcomes for eight of them have been previously reported [15]. All patients encountered one or more stimulation induced adverse effects, with the most reported being dysarthria (n = 6) and balance problems (n = 4).

#### 2.3.2 Literature

Bilateral DBS is the most reported surgical treatment for OT, with 23 patients included in this review, primarily case-reports. Of these, 16 patients received bilateral Vim-DBS and seven cZI-DBS. Three patients received unilateral Vim-DBS. All patients with bilateral DBS experienced substantial benefits, such as increased standing time, improved activities of daily living and/or a reduced unsteadiness. In contrast, patients with unilateral DBS experienced an initial benefit post-surgery, but this effect was not sustained over time. Among the 23 patients with bilateral DBS, adverse effects were reported in eight, and absence of adverse effects were reported in five patients. For the patients with unilateral DBS, two reported adverse effects.

In addition to the 23 DBS cases above, two patients transitioned from SCS to bilateral DBS (one with Vim-DBS and one with cZI-DBS) after an inadequate response to SCS; both patients experienced sufficient benefit from transition, with no adverse effects reported.

Of six patients with SCS included., four reported sufficient benefit and two reported a moderate effect. Of these six, two experienced no adverse effects, while two reported experiencing adverse effects. One patient subsequently transitioned to bilateral Vim-DBS, which yielded no additional benefit.

### 2.4 Other treatments

In the cohort from the Netherlands no other treatments were reported.

In the literature, one randomised, double blind, placebo controlled cross-over design trial assessed the safety and efficacy of botulinum toxin injected in the tibialis anterior bilaterally in eight OT patients, with no subjective or electrophysiological effects observed after 6 weeks. Various types of non-invasive stimulation treatments are documented. Repetitive transcranial magnetic stimulation (rTMS) (n = 19), *trans*-spinal direct current stimulation (n = 16), bilateral muscle tendon vibration (n = 9), and transcranial magnetic stimulation (n = 7), were reported to be effective in all or most patients, with observed improvements in balance, reduced tremor severity, decreased tremor amplitude and delayed tremor onset. Adverse effects were noted in four patients using bilateral muscle tendon vibration and in one patient receiving *trans*-spinal direct current stimulation. No adverse effects were reported in other studies.

Lumbar magnetic stimulation (n = 7), supramaximal nerve stimulation (n = 7), and submaximal nerve stimulation (n = 7), were reported to be not effective.

## 3 Discussion

Most commonly prescribed medications for OT are clonazepam, gabapentin, propranolol, primidone, and perampanel, with clonazepam most frequently cited in the literature and perampanel most commonly prescribed in the cohort from the Netherlands. Pharmacological treatments are often insufficiently effective for most individuals with OT, although not always discontinued. Also, patients might benefit from different or additional drugs, with most patients in the cohort from the Netherlands being on monotherapy. Perampanel appeared most effective in the Dutch cohort, while clonazepam appeared most effective in the literature. Adverse effects are common, particularly in perampanel, with data missing for nearly 90 % of patients in the systematic review. DBS, specifically targeting the Vim, was the most reported surgical intervention, showing sufficient benefit in both the cohort from the Netherlands and literature reports, though adverse effects were common.

Despite similar patient characteristics across populations, notable differences exist between the cohort from the Netherlands and literature reports concerning prescribed medications and pharmacological treatment outcomes. These disparities may result from selection bias influenced by several factors. Few trials have been conducted, with most knowledge derived from incomplete case reports and case-series that focus on current drug usage rather than long-term treatment outcomes. The Amsterdam UMC clinical OT-protocol excludes medications considered ineffective based on the literature, resulting in a narrower selection of prescribed drugs. Perampanel was often prescribed in patients who were referred after insufficient response to at least clonazepam and/or gabapentin, influenced by literature reports and positive stories on patients their social media OT-group. Limited studies exist on perampanel, especially for definitive EMG-confirmed primary OT. The largest study, involving 20 patients, was excluded due to incomplete EMG data [23]. This has led to an imbalance between the two populations regarding perampanel usage in particular. Moreover, patients who find clonazepam and gabapentin beneficial are unlikely to switch to perampanel, even if it could offer superior outcomes. This complicates comparisons. Despite limited treatment effectiveness, certain drugs, such as perampanel, show promising results, with benefits outweighing adverse effects.

Surgical interventions consistently showed sufficient benefit in both the cohort from the Netherlands and literature reports, though adverse effects were prevalent. Results were often obtained from more severely affected patients, using different reporting methods than pharmacological treatment studies, complicating comparisons. DBS appears more effective than SCS, however, the less invasive nature of SCS may influence treatment preferences. Also, novel techniques including sensing and adaptive stimulation might lead to increased tremor-reduction and less adverse effects. Given the invasive nature of surgical procedures, the reversibility of adverse effects in pharmacological treatment, sufficient relief in some patients, and unknown cost-benefit ratios, pharmacological options should be preferred, with surgery reserved as an alternative for medication-resistant patients.

Our study has several limitations. The OT survey questionnaires provides limited context and represent mere snapshots in time, potentially leading to reporting bias, as patients often switch medications due to varying effects. While we included data from 2014 to 2023 to mitigate this, bias remains possible. Patients without known tremor frequency were excluded because of uncertainty regarding primary OT diagnoses, limiting the amount of data, but the analysis was solely based on patients with confirmed primary OT. Furthermore, adverse effects were inconsistently reported, both in the literature and occasionally in our data, thereby potentially introducing bias. Study design differences, lack of control groups and blinding, differences in measuring and reporting effects, and potential publication bias in DBS and SCS cases also complicate comparisons. Lastly, the incomplete representation of all treatments described in the literature in our cohort complicates comparisons. Nonetheless, the most prescribed treatments were represented in both our cohort and the literature. Thereby providing an overview with valuable information about the different treatments and their effectiveness in OT, especially as randomized controlled trials are difficult to conduct in OT.

Based on current findings, perampanel and clonazepam are recommended, with perampanel showing higher efficacy but requiring further research through randomized trials to determine its (long-term) effectiveness compared to clonazepam. For medication-resistant patients, DBS remains the most promising surgical option. Randomized trials are, however, necessary to draw conclusions over DBS and SCS. Additionally, investigating other techniques, such as rTMS, is necessary since current evidence is insufficient to implement the technique in clinical practice.

This study provides a comprehensive overview of OT treatments options and their potential effectiveness. Pharmacological treatments are the most commonly used, followed by DBS and SCS, which show, despite limited experience, reasonable positive outcomes in medication refractory patients. Despite lack of robust trial data, pharmacological treatments, starting with either perampanel or clonazepam, are the preferred initial option, with reversable adverse effects and lower risk. The absence of randomized studies and reliance on indirect comparisons indicate the need for trials directly comparing perampanel with clonazepam. Additionally, data on mono versus combination therapy, and patient- and disease-characteristics related to treatment outcome are missing from the literature and should be addressed in future studies. Sham-controlled trials and blinding are necessary to draw conclusions on DBS and SCS, and investigating non-invasive stimulation techniques, a rapidly evolving field with potential in OT, is also warranted. Future studies should explore cost-benefit ratios, use diagnostic criteria for patient inclusion, and adopt standardized outcome measures like the OT-10 questionnaire to ensure consistent and reliable outcome assessment, and to increase reproducibility and comparability of results [24]. Overall, this study provides valuable insights into current OT treatment practices and their effectiveness, and forms a stepping stone for future studies.

## 4 Disclosure

*Funding sources and conflict of interests*:

All authors report no declarations of interest. This research did not receive any specific grant from funding agencies in the public, commercial, or not-for-profit sectors.

*Financial disclosure for the previous 12 months:*

Author M.M. and A.B. have none financial disclosures for the previous 12 months to report.

Author W.A. Babeliowsky received a grant from Stichting HET REMMERT ADRIAAN LAAN Fonds paid to the institution.

Author R.B. did not receive funding for the current work, the author received research grants Medtronic, Bial, ZonMw, AMC Foundation, ROMO Foundation, and Stichting ParkinsonFonds, all paid to the institution.

Author A.F. van Rootselaar has not received any funding related to the current research. The author received a research grant from Stichting De Merel, paid to the institution.

*Ethical Compliance Statement*:

We confirm that we have read the journal's position on issues involved in ethical publication and affirm that this work is consistent with those guidelines. Written informed consent was obtained for the data outside of standard patient care. A waiver of informed consent was obtained from the local medical ethical committee for the data that is part of standard patient care.

## CRediT authorship contribution statement

**W.A. Babeliowsky:** Writing – original draft, Resources, Methodology, Investigation, Conceptualization. **M.A. Meulepas:** Resources, Methodology. **A.W.G. Buijink:** Writing – review & editing, Methodology. **R.M.A. de Bie:** Writing – review & editing, Supervision. **A.F. van Rootselaar:** Writing – review & editing, Conceptualization.

## Declaration of competing interest

The authors declare that they have no known competing financial interests or personal relationships that could have appeared to influence the work reported in this paper.
