# Supplementary material for: How to treat orthostatic tremor – Cohort study and systematic review
Source: Clin Park Relat Disord. 2025 Apr 1;12:100318. doi: 10.1016/j.prdoa.2025.100318 (PMC12008538; doi:10.1016/j.prdoa.2025.100318)
Supplement: Supplementary Data 1 [file mmc1.docx]

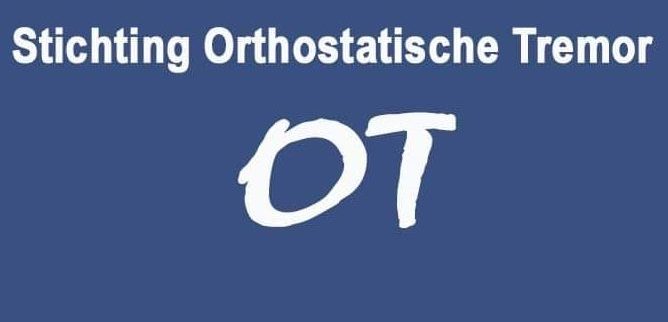


Orthostatische tremor survey

Name:..................................................

What is your gender................................F/M

Date of Birth:............................(dd/mm/year)

Date of completion: ............................(dd/mm/year)

Have you filled out the OT survey before: Yes / No / Don’t know

1. How severe have your OT symptoms been lately?

None Very severe

*Please mark a line on the scale above*

1. How much do the OT symptoms limit your daily life?

None _______________________________________________ Very severe

*Please mark a line on the scale above*

1. Which medication are you currently taking for tremors? Since when, and what is the effect??

* Rivotril/clonazepam - neurontin/gabapentin - fycompa/perampanel - propranolol - sinemet - mysoline/primidon - topiramaat - diazepam - seresta - vitamin B12 - or others.

| **Medication** | **since** | **Effect** (good/moderate/none) | **Side-effects** |
| --- | --- | --- | --- |
|  |  |  |  |
|  |  |  |  |
|  |  |  |  |
|  |  |  |  |

1. If a scientific study were to be conducted on the effect of medication for OT, would you be willing to:
   1. Temporarily stop your medication a few times?

- Yes/No

If yes, how long : a few days / a few weeks / doesn’t matter

- 1. Alternate your current medication with one you have taken before?
- Yes/No
  1. Alternate your current medication with one you haven’t taken before?
- Yes/No

**If you have previously filled out the OT survey, please report only changes below (you may leave blank what you have already filled out and remains the same).**

**Please complete as fully as possible, but you may skip questions if necessary.**

**If this is your first time filling out the survey, please answer questions 27 to 33. Thank you!**

1. Have you taken other medications for orthostatic tremor in the past?
   1. No
   2. Yes, namely:

| **Medication** | **From-to** | **Effect** (good/moderate/none) | **Side-effects** |
| --- | --- | --- | --- |
|  |  |  |  |
|  |  |  |  |
|  |  |  |  |
|  |  |  |  |

Rivotril/ clonazepam - neurontin/gabapentin   - fycompa/ perampanel -  propranolol   - sinemet -  mysoline/primidon -  topiramaat – diazepam - seresta   -of anders.

- 1. Why did you stop this medication?

1. Have you tried other therapies/aids, and what was the effect?

| **Therapy** | **since** | **Effect** (good/moderate/none) | **Side-effects** |
| --- | --- | --- | --- |
|  |  |  |  |
|  |  |  |  |
|  |  |  |  |

1. Have you been diagnosed with other/new conditions?

- No
- Yes, namely:

………………………………………………………………………

………………………………………………………………………

………………………………………………………………………

---------------------------------------------------------------------------------------------------------------------------

1. Do you experience tremors in the arms or other body parts?

- Yes, since …..

- No

1. Do you experience tremors in the legs/arms at rest?

     - Yes, since ….

- No

1. Does orthostatic tremor run in your family?

    - Yes, in …..

- No

1. Do you have symptoms that may suggest:

    - Parkinson’s disease

    - Parkinsonism

    - Restless legs

    - Essential tremor

    - Other movement disorder, namely:

11A) How long can you stand before you feel the tremor coming on?

     ----- seconds

     ----- minutes

11B) How long can you stand without support, without shifting or lifting your feet?

     ----- seconds

      ----- minutes

1. Do you play sports?

If yes, which?

1. Is walking going well?

      - Yes

- No

1. Do you sometimes fall?

      - Yes

- No
- Sometimes

1. Have you undergone DBS?

- Yes

- No
- If yes, in what year….

1. What are your experiences with DBS?
2. Are you considering DBS?

      - Yes

- Maybe
- No

1. Do you find OT progressive?

      - Yes

- No

1. Do other conditions affect your OT?

     - Stress

- Fatigue

     - Or other …..

1. Does weather affect your OT?

- Yes

- No

1. Do you experience sweating?

- Yes

- No

1. Do you receive assistance, such as in household or garden work, etc.?

      - Yes

- No

1. Can you shop independently?

      - Yes

- No

1. Can you travel independently?

     - Yes

- No

1. How is your general health?
2. How does OT affect your life?

**If this is your first time filling out the survey, please answer the following questions as well**

1. At what age did you first experience symptoms of orthostatic tremor?

.........Year

1. At what age were you diagnosed?

.........Year

1. How was the diagnosis made?

- Clinical examination (history + physical examination)

     - Stethoscope (helicopter sound)

- Muscle measurement (EMG) if known, tremor frequency: … Hz

1. What were the first OT symptoms you experienced?
2. Was further research done to investigate any underlying causes?

      - Yes

- No

If yes, what was investigated, and what was found?

1. In which hospital was the diagnosis made?
2. Do you consult a specialist after the diagnosis of OT?

- Yes

- No
