# Supplementary material for: How to treat orthostatic tremor – Cohort study and systematic review
Source: Clin Park Relat Disord. 2025 Apr 1;12:100318. doi: 10.1016/j.prdoa.2025.100318 (PMC12008538; doi:10.1016/j.prdoa.2025.100318)
Supplement: Supplementary Data 2 [file mmc2.docx]

Supplementary Table 1. Treatment effect Literature.

Abbreviations: EMG= electromyography, n.k.= not known

a= Duration of the disease from diagnosis till inclusion in the study

b= Data recorded from the m. tibialis anterior left and/or m. tibialis anterior right

*= Switched the DBS off

| **Medication** | **No. of cases** | **Treatment effect** | | |
| --- | --- | --- | --- | --- |
|  |  | **None** | **Sufficient benefit** | **Unknown** |
| ***Benzodiazepines*** |  |  |  |  |
| **Clonazepam** | 310 | 112 | 125 | 73 |
| **Diazepam** | 12 | 6 | 6 | - |
| **Lorazepam** | 6 | 3 | 3 | - |
| **Clorazepate** | 7 | 4 | 3 | - |
| **Alprazolam** | 7 | 5 | 2 | - |
| **Bromazepam** | 1 | - | 1 | - |
| **Chlordiazepoxide** | 2 | 2 | - | - |
| **Prazepam** | 1 | 1 | - | - |
| **All benzodiazepines** | 346 | 133 | 140 | 73 |
|  |  |  |  |  |
| ***Anticonvulsants*** |  |  |  |  |
| **Gabapentin** | 134 | 67 | 36 | 31 |
| **Valproate** | 49 | 38 | 10 | 1 |
| **Primidone** | 96 | 51 | 20 | 25 |
| **Topiramate** | 13 | 7 | 2 | 4 |
| **Pregabalin** | 12 | 6 | 2 | 4 |
| **Carbamazepine** | 7 | 5 | 1 | 1 |
| **Levetiracetam** | 10 | 5 | 1 | 4 |
| **Perampanel** | 10 | - | 4 | 6 |
| **Phenytoin** | 2 | 2 | - | - |
| **Lamotrigine** | 1 | 1 | - | - |
| **Tiagabine** | 1 | 1 | - | - |
| **Phenobarbital** | 7 | 4 | 3 | - |
| **All anticonvulsants** | 342 | 187 | 79 | 76 |
|  |  |  |  |  |
| ***β-Blockers*** |  |  |  |  |
| **Atenolol** | 4 | 2 | - | 2 |
| **Propranolol** | 84 | 50 | 20 | 14 |
| **Nadolol** | 3 | 1 | 2 | - |
| **β-Blocker** | 2 | 1 | - | 1 |
| **All β-Blocker** | 93 | 54 | 22 | 17 |
|  |  |  |  |  |
| ***Antiparkinsonian*** |  |  |  |  |
| **Biperiden** | 1 | 1 | - | - |
| **Bromocriptine** | 1 | 1 | - | - |
| **Levodopa/carbidopa** | 61 | 48 | 4 | 9 |
| **Levodopa/benserazide** | 6 | - | 2 | 4 |
| **Pramipexole** | 19 | 14 | 3 | 2 |
| **Ropinorole** | 3 | 2 | - | 1 |
| **Selegiline** | 5 | 4 | 1 | - |
| **Tolcapone** | 1 | 1 | - | - |
| **Pergolide** | 1 | - | 1 | - |
| **All antiparkinsonian** | 98 | 71 | 11 | 16 |
|  |  |  |  |  |
| **Antispasmodics** |  |  |  |  |
| **Baclofen** | 16 | 11 | 2 | 3 |
| **Methocarbomal** | 1 | - | 1 | - |
| **All antispasmodics** | 17 | 11 | 3 | 3 |
|  |  |  |  |  |
| **Anticholinergics** |  |  |  |  |
| **Trihexyphenidyl** | 9 | 7 | 1 | 1 |
| **Benztropine** | 1 | 1 | - | - |
| **Ethopropazine** | 1 | 1 | - | - |
| **All anticholinergics** | 11 | 9 | 1 | 1 |
|  |  |  |  |  |
| ***Antidepressants/anxiolytics*** |  |  |  |  |
| **Mirtazapine** | 8 | 7 | 1 | - |
| **Amitriptyline** | 3 | 3 | - | - |
| **Nortriptyline** | 2 | 2 | - | - |
| **Paroxetine** | 2 | 2 | - | - |
| **Sertraline** | 4 | 4 | - | - |
| **Venlafaxine** | 1 | 1 | - | - |
| **Bupropion** | 1 | 1 | - | - |
| **Buspirone** | 1 | 1 | - | - |
| **Fluoxetine** | 1 | 1 | - | - |
| **Etoperidone** | 1 | 1 | - | - |
| **All antidepressants/**  **anxiolytics** | 24 | 24 | 1 | 0 |
|  |  |  |  |  |
| ***Others*** |  |  |  |  |
| **Acetazolamide** | 8 | 5 | 2 | 1 |
| **Cyclo benzaprine** | 1 | 1 | - | - |
| **Levothyroxine** | 1 | - | - | 1 |
| **Pentoxifylline** | 1 | 1 | - | - |
| **Quinamm** | 1 | - | - | 1 |
| **Maxzide** | 1 | - | - | 1 |
| **Methazolamide** | 1 | 1 | - | - |
| **Verapamil** | 1 | 1 | - | - |
| **Zonisamide** | 2 | 2 | - | - |
| **All others** | 17 | 11 | 2 | 4 |

Abbreviations: No.= number of

- = No data available

Abbreviations: R= right, L= Left, DRT= Dendatorubrothalamic tract, ML= Medial Lemniscus, PT= Pyramidal Tract, n.k.= not known

When different settings are used for the right and left electrode it is indicated in the table using the mentioned abbreviations. Otherwise only one setting is give. In addition, the negative numbers indicate that the tract lies within the volume of activated tissue.
