# Supplementary material for: How to treat orthostatic tremor – Cohort study and systematic review
Source: Clin Park Relat Disord. 2025 Apr 1;12:100318. doi: 10.1016/j.prdoa.2025.100318 (PMC12008538; doi:10.1016/j.prdoa.2025.100318)
Supplement: Supplementary Data 3 [file mmc3.docx]

Supplementary Data

**Overview of included articles literature review**

*Case reports*

| **Nr.** | **Authors** | **Title** | **Journal** | **Year** | **Volume and page numbers** |
| --- | --- | --- | --- | --- | --- |
| 1 | Krauss JK, Weigel R, Blahak C, et al. | Chronic spinal cord stimulation in medically intractable orthostatic tremor. | Journal of Neurology, Neurosurgery and Psychiatry | 2006 | 77(9):1013-1016 |
| 2 | Magarinos-Ascone C, Ruiz FM, Millan AS, et al. | Electrophysiological evaluation of thalamic DBS for orthostatic tremor. | Movement Disorders | 2010 | 25(14):2476-2477 |
| 3 | Lyons MK, Behbahani M, Boucher OK, Caviness JN, Evidente VG. | Orthostatic tremor responds to bilateral thalamic deep brain stimulation. | Tremor and Other Hyperkinetic Movement disorders | 2012 | 2 |
| 4 | Milone M, Klassen BT, Landsverk ML, Haas RH, Wong LJ. | Orthostatic tremor, progressive external ophthalmoplegia, and Twinkle. | JAMA Neurology | 2013 | 70(11):1429-1431 |
| 5 | Bhattacharyya KB, Das D. | Familial orthostatic tremor and essential tremor in two young brothers: A rare entity. | Annals of Indian Academy of Neurology | 2013 | 16(2):276-278 |
| 6 | Virmani T, Louis ED, Waters C, Pullman SL. | Familial orthostatic tremor: an additional report in siblings. | Neurology | 2012 | 79(3):288-289 |
| 7 | Jones L, Bain PG. | Orthostatic tremor. | Practical Neurology | 2011 | 11(4):240-243 |
| 8 | Yaltho TC, Ondo WG. | Thalamic deep brain stimulation for orthostatic tremor. | Tremor and Other Hyperkinetic Movement disorders | 2011 | 1 |
| 9 | Littmann L. | Fact or artifact? The electrocardiographic diagnosis of orthostatic tremor. | Journal of Electrocardiology | 2010 | 43(3):270-273 |
| 10 | Ramtahal J, Larner AJ. | Shaky legs? Think POT! | Age and Ageing | 2009 | 38(3):352-353 |
| 11 | Wegner F, Strecker K, Boeckler D, et al. | Intact serotonergic and dopaminergic systems in two cases of orthostatic tremor. | Journal of Neurology | 2008 | 255(11):1840-1842 |
| 12 | Espay AJ, Duker AP, Chen R, et al. | Deep brain stimulation of the ventral intermediate nucleus of the thalamus in medically refractory orthostatic tremor: preliminary observations. | Movement Disorders | 2008 | 23(16):2357-2362 |
| 13 | Guridi J, Rodriguez-Oroz MC, Arbizu J, et al. | Successful thalamic deep brain stimulation for orthostatic tremor. | Movement Disorders | 2008 | 23(13):1808-1811 |
| 14 | Fischer M, Kress W, Reiners K, Rieckmann P. | Orthostatic tremor in three brothers. | Journal of Neurology | 2007 | 254(12):1759-1760 |
| 15 | Contarino MF, Welter ML, Agid Y, Hartmann A. | Orthostatic tremor in monozygotic twins. | Neurology | 2006 | 66(10):1600-1601 |
| 16 | Bacsi AM, Halmagyi GM, Colebatch JG. | Sway patterns in a case of orthostatic tremor responsive to alcohol. | Movement Disorders | 2004 | 19(12):1459-1463 |
| 17 | Myers BH, Scott BL. | A case of combined orthostatic tremor and primary gait ignition failure. | Clinical Neurology and Neurosurgery | 2003 | 105(4):277-280 |
| 18 | Bhattacharyya KB, Basu S, Roy AD, Bhattacharya S. | Orthostatic tremor: report of a case and review of the literature. | Neurology India | 2003 | 51(1):91-93 |
| 19 | Finkel MF. | Pramipexole is a possible effective treatment for primary orthostatic tremor (shaky leg syndrome). | Archives of neurology | 2005 | 57(10):1519-1520 |
| 20 | Trip SA, Wroe SJ. | Primary orthostatic tremor associated with a persistent cerebrospinal fluid monoclonal IgG band. | Movement Disorders | 2003 | 18(3):345-346 |
| 21 | Sander HW, Masdeu JC, Tavoulareas G, Walters A, Zimmerman T, Chokroverty S. | Orthostatic tremor: an electrophysiological analysis. | Movement Disorders | 1998 | 13(4):735-738 |
| 22 | Hanna M, Mills K, Pazdera L, Newsom-Davis J. | Primary orthostatic tremor with prominent muscle hypertrophy. | Neurology | 1997 | 49(3):872-874 |
| 23 | Cabrera-Valdivia F, Jimenez-Jimenez FJ, Garcia Albea E, Tejeiro-Martinez J, Vaquero Ruiperez JA, Ayuso-Peralta L. | Orthostatic tremor: successful treatment with phenobarbital. | Clinical Neuropharmacology | 1991 | 14(5):438-441 |
| 24 | Sanitate SS, Meerschaert JR. | Orthostatic tremor: delayed onset following head trauma. | Archives of Physical Medicine and Rehabilitation | 1993 | 74(8):886-889 |
| 25 | Yague S, Veciana M, Pedro J, Campdelacreu J. | Importance of electromyographic studies in the diagnosis of orthostatic tremor. | Neurologia | 2011 | 26(1):53-55 |
| 26 | Contarino MF, Bour LJ, Schuurman PR, et al. | Thalamic deep brain stimulation for orthostatic tremor: Clinical and neurophysiological correlates. | Parkinsonism & Related Disorders | 2015 | 21(8):1005-1007 |
| 27 | Coleman RR, Starr PA, Katz M, et al. | Bilateral Ventral Intermediate Nucleus Thalamic Deep Brain Stimulation in Orthostatic Tremor. | Stereotactic and Functional Neurosurgery | 2016 | 94(2):69-74 |
| 28 | Vidailhet M, Roze E, Maugest L, Gallea C. | Lessons I have learned from my patients: everyday life with primary orthostatic tremor. | Journal of Clinical Movement Disorders | 2017 | 4:1 |
| 29 | Chiang HL, Tai YC, McMaster J, Fung VS, Mahant N. | Primary orthostatic tremor: is deep brain stimulation better than spinal cord stimulation? | Journal of Neurology, Neurosurgery and Psychiatry. | 2017 | 88(9):804-805 |
| 30 | Lehn AC, O'Gorman C, Olson S, Salari M. | Thalamic Ventral Intermediate Nucleus Deep Brain Stimulation for Orthostatic Tremor. | Tremor and Other Hyperkinetic Movement disorders | 2017 | 7:479 |
| 31 | Grobe-Einsler M, Kaut O. | Remarkable improvement of primary orthostatic tremor using perampanel. | Neurological Research and Practice | 2020 | 2:3 |
| 32 | Verriello L, Bernardini A, Pauletto G, et al. | Primary Orthostatic Tremor in 2 Siblings. | Movement Disorders Clinical Practice | 2020 | 7(2):234-236 |
| 33 | Wadhwa A, Schaefer SM. | Successful Treatment of Primary Orthostatic Tremor Using Perampanel. | Tremor and Other Hyperkinetic Movement disorders | 2019 | 9 |
| 34 | Ruiz-Julian M, Orozco JL, Gironell A. | Complete Resolution of Symptoms of Primary Orthostatic Tremor with Perampanel. | Tremor and Other Hyperkinetic Movement disorders | 2081 | 8:552 |
| 35 | Athauda D, Georgiev D, Aviles-Olmos I, et al | Thalamic-Caudal Zona Incerta Deep Brain Stimulation for Refractory Orthostatic Tremor: A Report of 3 Cases. | Movement Disorders Clinical Practice | 2017 | 4(1):105-110 |
| 36 | Pintea B, de Boni L, Kinfe TM. | Subperceptional Burst Versus Perceptional Tonic Spinal Cord Stimulation Waveforms for Drug-resistant Orthostatic Tremor: Comparative Data of 2 Cases. | Movement Disorders Clinical Practice | 2017 | 4(4):612-615 |
| 37 | Poersch M. | Orthostatic tremor: combined treatment with primidone and clonazepam. | Movement Disorders | 1994 | 9(4):467 |
| 38 | Britton TC, Thompson PD, van der Kamp W, et al. | Primary orthostatic tremor: further observations in six cases. | Journal of Neurology | 1992 | 239(4):209-217 |
| 39 | Santus G, Faletti S, Grandis D. | Orthostatic tremor and behavioral frontotemporal dementia: a case report with 7 years of follow-up. | Neurological Sciences | 2019 | 40(11):2415-2417 |
| 40 | Vetrugno R, Fabbri M, Antelmi E, D'Angelo R, Rinaldi R. | Orthostatic tremor heralding the onset of stiff-person syndrome. | Neurology | 2013 | 81(15):1361-1362 |
| 41 | FitzGerald PM, Jankovic J. | Orthostatic tremor: an association with essential tremor. | Movement Disorders | 1991 | 6(1):60-64 |
| 42 | Uncini A, Onofrj M, Basciani M, Cutarella R, Gambi D. | Orthostatic tremor: report of two cases and an electrophysiological study. | Acta Neurologica Scandinavica | 1989 | 79(2):119-122 |
| 43 | Wiendels NJ, Tromp SC. | [Orthostatic tremor: unsteadiness while standing still]. | Nederlands Tijdschrift voor Geneeskunde | 2012 | 156(21):A4396 |
| 44 | Swinnen B, de Bie RMA, van Rootselaar AF. | [Orthostatic tremor]. | Nederlands Tijdschrift voor Geneeskunde | 2021 | 165 |
| 45 | Thompson PD, Rothwell JC, Day BL, Berardelli A, Dick JP, Kachi T, Marsden CD. | The physiology of orthostatic tremor | Archives of neurology | 1986 | 43(6):584-7 |

*Case-series*

| **Nr.** | **Authors** | **Title** | **Journal** | **Year** | **Volume and page numbers** |
| --- | --- | --- | --- | --- | --- |
| 1 | Hassan A, Ahlskog JE, Matsumoto JY, Milber JM, Bower JH, Wilkinson JR. | Orthostatic tremor: Clinical, electrophysiologic, and treatment findings in 184 patients. | Neurology | 2016 | 86(5):458-464 |
| 2 | Gilmore G, Murgai A, Nazer A, Parrent A, Jog M. | Zona incerta deep-brain stimulation in orthostatic tremor: efficacy and mechanism of improvement. | Journal of Neurology | 2019 | 266(11):2829-2837 |
| 3 | Blahak C, Sauer T, Baezner H, et al. | Long-term follow-up of chronic spinal cord stimulation for medically intractable orthostatic tremor. | Journal of Neurology | 2016 | 263(11):2224-2228 |
| 4 | Hewitt AL, Klassen BT, Lee KH, Van Gompel JJ, Hassan A. | Deep brain stimulation for orthostatic tremor: A single-center case series. | Neurology Clinical Practice | 2020 | 10(4):324-332 |
| 5 | Mestre TA, Lang AE, Ferreira JJ, et al. | Associated movement disorders in orthostatic tremor. | Journal of Neurology, Neurosurgery and Psychiatry | 2012 | 83(7):725-729 |
| 6 | Boroojerdi B, Ferbert A, Foltys H, Kosinski CM, Noth J, Schwarz M. | Evidence for a non-orthostatic origin of orthostatic tremor. | Journal of Neurology, Neurosurgery and Psychiatry | 1999 | 66(3):284-288 |
| 7 | Gerschlager W, Katzenschlager R, Schrag A, et al. | Quality of life in patients with orthostatic tremor. | Journal of Neurology | 2003 | 250(2):212-215 |
| 8 | Spiegel J, Fuss G, Krick C, Dillmann U. | Impact of different stimulation types on orthostatic tremor. | Clinical Neurophysiology | 2004 | 115(3):569-575 |
| 9 | Swinnen B, de Waal H, Buijink AWG, de Bie RMA, van Rootselaar AF. | The Phenomenology of Primary Orthostatic Tremor. | Movement Disorders Clinical Practice | 2022 | 9(4):489-493 |
| 10 | Bicart-See L, Thibault JL, Poujois A, et al. | Associated co-morbidities in a retrospective cohort of orthostatic tremor. | Journal of Neurology | 2021 | 268(2):467-473 |
| 11 | Bhatti DE, Thompson RJ, Malgireddy K, et al. | Anxiety spectrum disorders are common in patients with orthostatic tremor. | Clinical Parkinsonism & Related Disorders | 2019 | 1:10-12 |
| 12 | Opri E, Hu W, Jabarkheel Z, et al. | Gait characterization for patients with orthostatic tremor. | Parkinsonism & Related Disorders | 2020 | 71:23-27 |
| 13 | Wuehr M, Schlick C, Mohwald K, Schniepp R. | Proprioceptive muscle tendon stimulation reduces symptoms in primary orthostatic tremor. | Journal of Neurology | 2018 | 265(7):1666-1670 |
| 14 | Maugest L, McGovern EM, Mazalovic K, et al. | Health-Related Quality of Life Is Severely Affected in Primary Orthostatic Tremor. | Frontiers in Neurology | 2017 | 8:747 |
| 15 | Wu YR, Ashby P, Lang AE. | Orthostatic tremor arises from an oscillator in the posterior fossa. | Movement Disorders | 2001 | 16(2):272-279 |
| 16 | Feil K, Bottcher N, Guri F, et al. | Long-term course of orthostatic tremor in serial posturographic measurement. | Parkinsonism & Related Disorders | 2015 | 21(8):905-910 |
| 17 | Trocello JM, Zanotti-Fregonara P, Roze E, et al. | Dopaminergic deficit is not the rule in orthostatic tremor. | Movement Disorders | 2008 | 23(12):1733-1738 |
| 18 | Rodrigues JP, Edwards DJ, Walters SE, et al. | Gabapentin can improve postural stability and quality of life in primary orthostatic tremor. | Movement Disorders | 2005 | 20(7):865-870 |

*Case-control studies*

| **Nr.** | **Authors** | **Title** | **Journal** | **Year** | **Volume and page numbers** |
| --- | --- | --- | --- | --- | --- |
| 1 | Gallea C, Popa T, Garcia-Lorenzo D, et al. | Orthostatic tremor: a cerebellar pathology? | Brain | 2016 | 139(Pt 8):2182-2197 |
| 2 | McAuley JH, Britton TC, Rothwell JC, Findley LJ, Marsden CD. | The timing of primary orthostatic tremor bursts has a task-specific plasticity. | Brain | 2000 | 123(Pt 2):254-266 |
| 3 | Fung VS, Sauner D, Day BL | A dissociation between subjective and objective unsteadiness in primary orthostatic tremor. | Brain | 2001 | 124(Pt 2):322-330 |
| 4 | Bacsi AM, Fung VS, Colebatch JG. | Sway patterns in orthostatic tremor: impairment of postural control mechanisms. | Movement Disorders | 2005 | 20(11):1469-1475 |
| 5 | Schoberl F, Feil K, Xiong G, et al. | Pathological ponto-cerebello-thalamo-cortical activations in primary orthostatic tremor during lying and stance. | Brain | 2017 | 140(1):83-97 |
| 6 | Thompson R, Bhatti DE, Hellman A, et al. | Ataxia Prevalence in Primary Orthostatic Tremor. | Tremor and Other Hyperkinetic Movement disorders | 2020 | 10:54 |
| 7 | Katzenschlager R, Costa D, Gerschlager W, et al. | [123I]-FP-CIT-SPECT demonstrates dopaminergic deficit in orthostatic tremor. | Annals of Neurology | 2003 | 53(4):489-496 |

*Trials*

| **Nr.** | **Authors** | **Title** | **Journal** | **Year** | **Volume and page numbers** |
| --- | --- | --- | --- | --- | --- |
| 1 | Bertram K, Sirisena D, Cowey M, Hill A, Williams DR. | Safety and efficacy of botulinum toxin in primary orthostatic tremor. | Journal of Clinical Neuroscience | 2013 | 20(11):1503-1505 |
| 2 | Onofrj M, Thomas A, Paci C, D'Andreamatteo G. | Gabapentin in orthostatic tremor: results of a double-blind crossover with placebo in four patients. | Neurology | 1998 | 51(3):880-882 |
| 3 | Lamy JC, Varriale P, Apartis E, et al. | Trans-Spinal Direct Current Stimulation for Managing Primary Orthostatic Tremor. | Movement Disorders | 2021 | 36(8):1835-1842 |
| 4 | Hu W, Legacy J, Ferng A, Wagle Shukla A. | Potential role for rTMS in treating Primary Orthostatic Tremor. | Brain Stimulation | 2020 | 13(4):1105-1107 |
